# Large-scale analysis of the ARF and Aux/IAA gene families in 406 horticultural and other plants

**DOI:** 10.1186/s43897-024-00090-7

**Published:** 2024-04-09

**Authors:** Shuyan Feng, Nan Li, Huilong Chen, Zhuo Liu, Chunjin Li, Rong Zhou, Yingchao Zhang, Rui Cao, Xiao Ma, Xiaoming Song

**Affiliations:** 1 School of Life Sciences/Library, North China University of Science and Technology, Tangshan, Hebei 063210 China; 2 College of Grassland Science and Technology, China Agricultural University, Beijing, 100193 China; 3 Department of Food Science, Aarhus University, Aarhus, 8200 Denmark; 4 College of Horticultural Science & Technology, Hebei Normal University of Science & Technology, Qinhuangdao, Hebei 066600 China

**Keywords:** ARF, Aux/IAA, Large-scale analysis, Evolutionary origin, Duplication type

## Abstract

The auxin response factor (ARF) and auxin/indole-3-acetic acid (Aux/IAA) family of genes are central components of the auxin signaling pathway and play essential roles in plant growth and development. Their large-scale analysis and evolutionary trajectory of origin are currently not known. Here, we identified the corresponding ARF and Aux/IAA family members and performed a large-scale analysis by scanning 406 plant genomes. The results showed that the ARF and Aux/IAA gene families originated from charophytes. The ARF family sequences were more conserved than the Aux/IAA family sequences. Dispersed duplications were the common expansion mode of ARF and Aux/IAA families in bryophytes, ferns, and gymnosperms; however, whole-genome duplication was the common expansion mode of the ARF and Aux/IAA families in basal angiosperms, magnoliids, monocots, and dicots. Expression and regulatory network analyses revealed that the *Arabidopsis thaliana* ARF and Aux/IAA families responded to multiple hormone, biotic, and abiotic stresses. The APETALA2 and serum response factor-transcription factor gene families were commonly enriched in the upstream and downstream genes of the ARF and Aux/IAA gene families. Our study provides a comprehensive overview of the evolutionary trajectories, structural functions, expansion mechanisms, expression patterns, and regulatory networks of these two gene families.

**Supplementary Information:**

The online version contains supplementary material available at 10.1186/s43897-024-00090-7.

## Core

Our large-scale analysis revealed that ARF and Aux/IAA gene families originated from charophytes. The identification of duplication types revealed the expansion of the two families in different plant taxa. Our study determined the origin and molecular evolution of these two families and provides a theoretical basis for crop trait improvement.

## Gene & accession numbers

All sources of sequence data used in this article can be found in Supplementary Table 1. All the gene information is provided in our Plant Hormone Gene Database (PHGD: http://phgd.bio2db.com).

## Introduction

Auxin is one of the most important plant hormones that regulate plant growth and development (Cakir et al. 2013). It is involved in the regulation of many physiological processes in plants and has important roles in cell differentiation, seed growth, lateral rhizogenesis in roots, apical dominance, and fruit development (Li et al. 2016, 2022; Khan et al. 2024). Auxin response factors (ARFs) and auxin/indole-3-acetic acids (Aux/IAAs) are the two main transcription factors that are involved in auxin regulation/response (Hagen and Guilfoyle 2002; Li et al. 2023).

ARFs can regulate gene expression by binding to auxin response elements (TGTCTC, TGTCCC, TGTCAC, and TGTCGG) in the auxin response genes (Liscum and Reed 2002). Aux/IAA proteins are short-lived nuclear proteins with half-lives ranging from 10 min for IAA7/IAA17 to 60 min for IAA28 in *Arabidopsis*. These proteins are involved in various developmental stages of plants and are important growth factors (Gray et al. 2001; Ramos et al. 2001). At low levels of auxin, Aux/IAA proteins form dimers with ARFs, which inhibit ARF activity by recruiting the synergistic blocker TOPLESS, preventing ARF proteins from regulating the transcription of downstream genes, and thus interrupting the auxin synthesis pathway (Ulmasov et al. 1999; Guilfoyle and Hagen 2007). At high levels of auxin, the ABA-responsive fba domain-containing protein and 26S proteasomes promote Aux/IAA ubiquitination and degradation, releasing ARFs to bind to the promoters of auxin-responsive genes and regulate the transcription of downstream genes (Ulmasov et al. 1999; Guilfoyle and Hagen 2007).

In evolutionary order, the known plant species on Earth can be classified as algae, bryophytes, ferns, gymnosperms, basal angiosperms, magnoliids, monocots, and dicots (Goodstein et al. 2012; Song et al. 2021b; Yu et al. 2022a). The continuous development of sequencing technologies has led to the generation of high-quality plant genome sequences, which has made it possible to explore the origin and evolution of gene families in a comprehensive manner. Currently, ARF and Aux/IAA have been identified and studied in several single species, e.g., *Arabidopsis thaliana* (Abel et al. 1995; Okushima et al. 2005), *Brassica rapa* (Huang et al. 2016), *Fragaria vesca* (Wang et al. 2019; Su et al.^ 2021^a), *Populus trichocarpa* (Kalluri et al. 2007), *Malus domestica* (Luo et al. 2014; Su et al. 2021b), *Medicago truncatula* (Shen et al. 2015; Liu et al. 2021), *Oryza sativa* (Jain et al. 2006; Wang et al. 2007), *Zea mays* (Liu et al. 2011; Xu et al. 2021), and *Hordeum vulgare* (Tombuloglu 2019; Shi et al. 2020). Studies of the origin of ARF and Aux/IAA in a limited number of species have suggested that ARF may have originated from charophytes, while Aux/IAA can be traced back to the ancestors of land plants and green algae. As more plant genomes are released, the origin and evolution of ARF and Aux/IAA may be updated.

Therefore, we collected high-quality plant genomic data to analyze the origin, evolutionary trajectory, expansion mechanisms, expression patterns, and regulatory networks of the ARF and Aux/IAA gene families at the large-scale level. Our study explored the origin and molecular evolution of these two families and the results provide a theoretical basis for crop trait improvement.

## Results

### Identification and distribution of gene family members in each plant taxon

We identified ARF and Aux/IAA family members from 406 species, including glaucophytes (1), prasinodermophytes (1), red algae (7), green algae (22), charophytes (7), bryophytes (8), pteridophytes (5), gymnosperms (8), basal angiosperms (3), magnoliids (5), monocots (81), and dicots (258) (Table S1). We counted the number of ARF and Aux/IAA family members of different plant taxa (Table S2). Among the algal plant taxa, ARF and Aux/IAA family members were present only in charophytes. The ARF family members were distributed in one chlorokybophyte, one charophyte, and two zygnematophytes, and the Aux/IAA family members were distributed in one charophyte and one zygnematophyte. These results suggest that the origin of the ARF and Aux/IAA families can be traced back to the charophyte period.

There were more ARF family members than Aux/IAA family members in bryophytes, while the opposite was true for most species of other plant taxa (Table S2). The maximum number of ARF family members was 15 in *Physcomitrella patens,* and the minimum was three in *Anthoceros agrestis*; the maximum number of Aux/IAA family members was four in *P. patens*, and the minimum was one in *A. agrestis*. Among the ferns, the maximum number of ARF family members was 21 (*Alsophila spinulosa*), the minimum was seven (*Selaginella moellendorffii* and *Isoetes taiwanensis*); the maximum number of Aux/IAA family members was 19 (*A. spinulosa*), and the minimum was nine (*Azolla filiculoides* and *Salvinia cucullata*) (Table S2 ). Among gymnosperms, the maximum number of ARF family members was 18 (*Cycas panzhihuaensis*), the minimum was five (*Pinus taeda*); the maximum number of Aux/IAA family members was 35 (*Abies alba*), and the minimum was six (*Gnetum montanum*) (Table S2). Among the basal angiosperms, the maximum number of ARF family members was 47 (*Euryale ferox*), the minimum was 16 (*Nymphaea colorata*); the maximum number of Aux/IAA family members was 59 (*E. ferox*), and the minimum was 21 (*Amborella trichopoda*) (Table S2). Among the magnoliids, the maximum number of ARF family members was 23 (*Magnolia biondii* and *Cinnamomum kanehirae*), the minimum was 13 (*Aristolochia contorta* and *Aristolochia fimbriata*); and the maximum number of Aux/IAA family members was 24 (*M. biondii*), while the minimum was 13 (*A. fimbriata*). Among the monocots, the maximum number of ARF family members was 129 (*Dendrocalamus latiflorus*), the minimum was 10 (*Lemna minuta*); the maximum number of Aux/IAA family members was 200 (*D. latiflorus*), and the minimum was 12 (*L. minuta*, *Apostasia shenzhenica* and *Gastrodia menghaiensis*) (Table S2). Among the dicots, the maximum number of ARF family members was 96 (*Helianthus annuus*), the minimum was four (*Corylus avellana*); the maximum number of Aux/IAA family members was 115 (*Brassica napus*), and the minimum was 8 (*Amaranthus hypochondriacus*) (Table S2).

### Phylogenetic analysis and exploration of the evolutionary trajectory

**Fig. 1:**
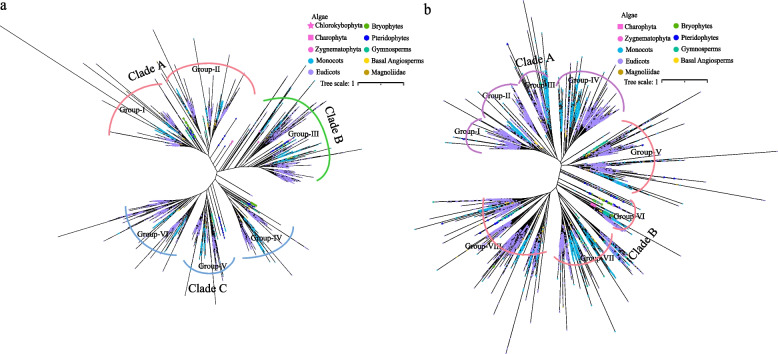
Phylogenetic analysis of ARF and Aux/IAA family genes in 406 plants. **a** Construction of a phylogenetic tree using the protein sequences of all the ARF family genes from 406 species. **b** Construction of a phylogenetic tree using the protein sequences of all the Aux/IAA family genes from 406 species

To explore the phylogenetic relationships between the ARF and Aux/IAA gene families in plant taxa, we constructed phylogenetic trees of 406 species, encompassing the ARF and Aux/IAA families (Fig. 1).

Based on the topology, the ARF gene was divided into clade A, clade B, and clade C (Fig. 1a). Clade A was further divided into group I and group II, and clade C was further divided into group IV, group V, and group VI. We found that the ARF genes of Chlorokybophyta and Charophyta were clustered in group III (clade B), while the ARF genes of Zygnematophyta were clustered in group II (clade A). The ARF genes of the charophyte were clustered into group I (clade A), group III (clade B), and group IV (clade C). The ARF genes of bryophytes, ferns, and gymnosperms were clustered into all subgroups (group I, group II, group III, group IV, and group V) except group VI (clade C). The ARF genes of basal angiosperms, magnoliids, monocots, and dicots were clustered into all six groups.

The Aux/IAA phylogenetic tree was divided into clade A and clade B (Fig. 1b). Clade A was further divided into group I, group II, group III, and group IV, and clade B was further divided into group V, group VI, group VII, and group VIII. The results showed that the Aux/IAA genes of Charophyta and Zygnematophyta were clustered into group VI (clade B). The Aux/IAA genes of the bryophytes were clustered into group VI (clade B) and group VIII (clade B). The Aux/IAA genes of the ferns were clustered into all eight groups. The Aux/IAA genes of the gymnosperms were clustered into group III (clade A), group IV (clade A), group V (clade B), group VI (clade B), group VII (clade B), and group VIII (clade B). The Aux/IAA genes of basal angiosperms and magnoliids were clustered into all subgroups (group II, group III, group IV, group V, group VI, group VII, and group VIII) except group I (clade A). The Aux/IAA genes of monocots and dicots were clustered into all eight groups.

**Fig. 2:**
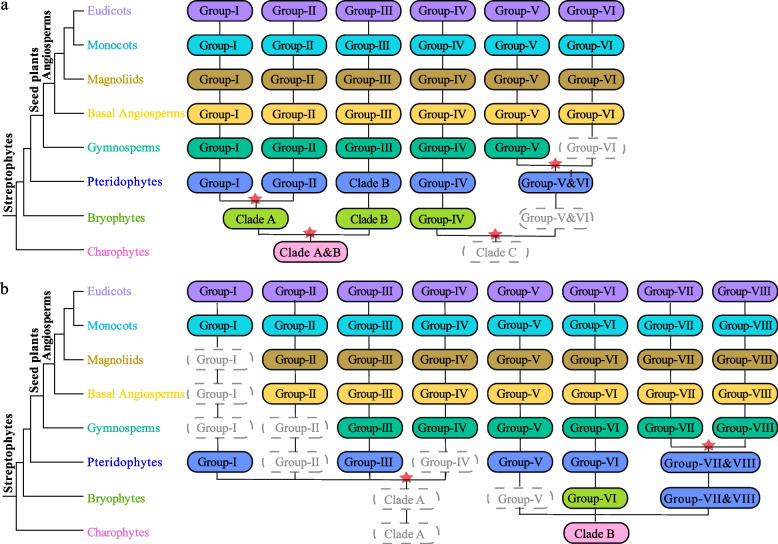
The ancient evolutionary trajectory of ARF and Aux/IAA family genes in plants. **a** The evolutionary trajectory of ARF genes in plants. **b** The evolutionary trajectory of Aux/IAA genes in plants. The eight major plant lineages are represented by different colors’ solid round rectangles indicate the presence of ARF and Aux/IAA family genes in the corresponding plant lineages, and dashed rectangles suggest the absence of genes due to gene losses. Inferred ancient gene duplications are depicted as red pentacles

To explore the evolutionary history of different groups of family genes in the phylogenetic tree in more depth, the evolutionary trajectories of the ARF and Aux/IAA families in different lineages of plants were schematically mapped according to the phylogenetic tree and species relationships (Fig. 2).

The evolutionary trajectory of the ARF gene family in the plant lineage is shown in Fig. 2a. We hypothesized that the ARF family genes were initially a class in the charophyte and were named clade A and clade B. The ARF family genes were separated into clade A and clade B genes at the bryophyte stage, possibly due to gene duplication. Later, it reached the fern stage, clade A differentiated into group I and group II, while clade B did not undergo differentiation. Then, starting from ferns, the three grouping relationships that diverged continued to be maintained in subsequent higher plant lineages. In contrast, the missing clade C in the charophytes may be due to a lack of data or a loss of genes. At the bryophyte stage, clade C likely diverged into group IV and continued to form more plant lineages. Groups V and VI included bryophytes, which were missing in charophytes but appeared in ferns. Until the gymnosperm lineage was reached, group V diverged and continued to form higher plant lineages. Group VI included genes that were missing from gymnosperms but appeared in basal angiosperms and continued to include more plant lineages.

Similarly, we explored the evolutionary trajectory of the Aux/IAA gene family in the plant lineage (Fig. 2b). Initially, only clade B was present among the charophytes. During the emergence of the bryophytes, clade B diverged into group VI and groups VII and VIII. Group VI continued to generate additional plant lineages, while groups VII and VIII contributed to the development of the fern lineage. In the gymnosperm lineage, groups VII and group VIII further diverged and continued to form more plant lineages. Group V first occurred in ferns, but continued to develop into more advanced plant lineages. However, clade A was missing from charophytes and bryophytes, and was first found in ferns. Groups I and III of clade A first appeared in the ferns, and group III continued into the higher plant lineages. Interestingly, group I was lost in the gymnosperm, basal angiosperm, and magnoliid lineages, but was retained in the more advanced monocot and dicot lineages. Group IV was first detected in the gymnosperm lineage, and group II was first detected in the basal angiosperm lineage.

In conclusion, the analysis of the evolutionary patterns of the ARF and Aux/IAA gene families across plant taxa contributed to a better understanding of the phylogenetic relationships and evolutionary history of the two families, which reflects the need for and advantages of a large-scale family gene analysis covering the entire plant taxon.

### Conserved motif identification and distribution

Conservative motif analysis is important for revealing the conserved patterns of gene families. Therefore, a total of 18 representative species from lower to higher plants were selected for analysis, including dicot (*A. thaliana*), monocot (*O. sativa*), basal angiosperm (*A. trichopoda*), magnoliid (*A. fimbriata*), gymnosperm (*Picea abies*), fern (*S. moellendorffii*), all bryophytes (eight in total, including *Marchantia polymorpha*, *P. patens*, and *Fontinalis antipyretica*), and algal species (for ARF, four charophyte species [*Chlorokybus atmophyticus*, *Chara braunii*, *Mesotaenium endlicherianum*, and *Spirogloea muscicola*]; for Aux/IAA, two charophyte species [*C. braunii* and *Penium margaritaceum*]).

**Fig. 3:**
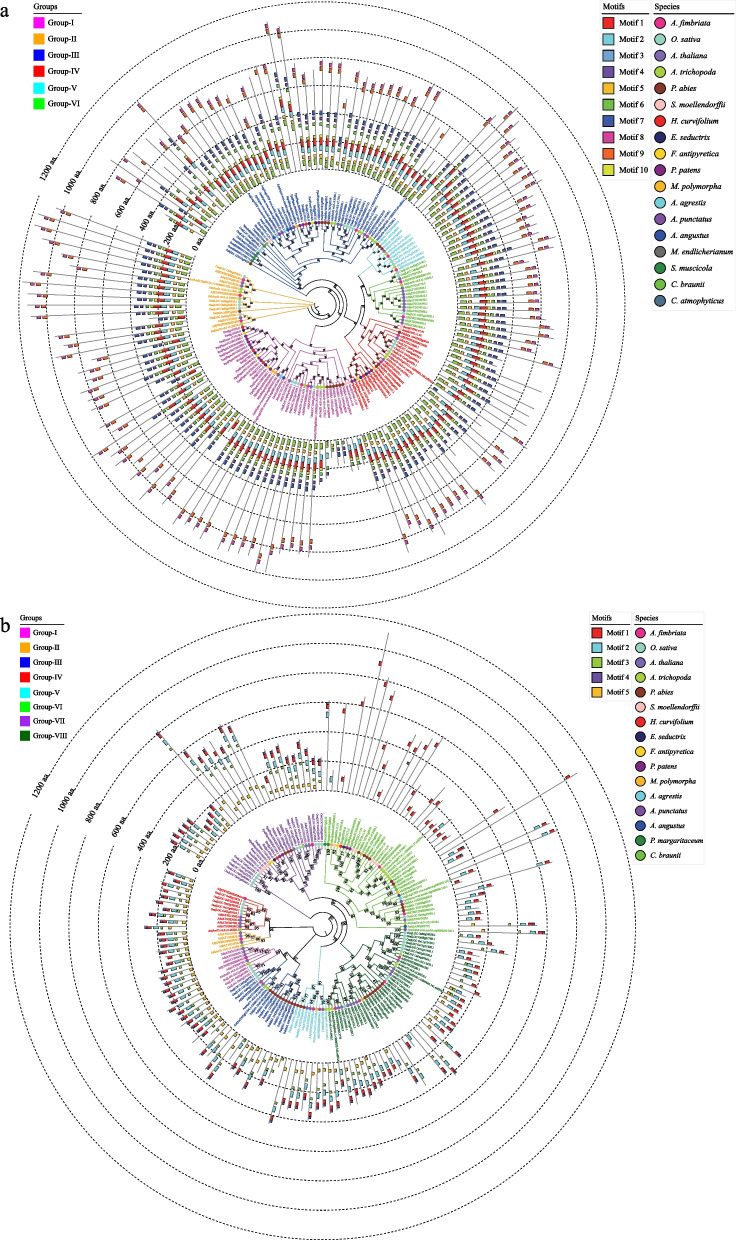
Phylogenetic and conversed motif analyses of ARF and Aux/IAA family genes from representative species. **a** Phylogenetic and conversed motif analyses of ARF family genes from 18 representative species. **b** Phylogenetic and conversed motif analyses of Aux/IAA family genes from 16 representative species. A phylogenetic tree was constructed using FastTree software. The motifs were identified using the MEME program

Ten and five motifs were identified in ARF and Aux/IAA family proteins, respectively (Fig. 3). Eight of the ARF family motifs (motifs 1 − 7 and motif 10) were present in almost all the ARF proteins. However, these conserved motifs were also lost in some ARF proteins, such as Pab|MA10199543g0020 and Pab|MA10121946g0020 in group I, Pab|MA85955g0020 in group II, Atr|AmTrv6.0c4.8580.1 in group III, Atr|AmTrv6.0c1.18630.1 in group IV, and Ath|AT1G34170.3 in group VI. This indicated that the conserved motifs of ARFs were lost in some proteins during plant evolution. Notably, the 10 conserved motifs of the ARFs in group V, which were the most conserved relative to the other groups, were completely preserved.

In the Aux/IAA family, motifs 1 − 5 were present in almost all Aux/IAA proteins except for group VI. However, some motifs were also lost in some proteins, such as Ath|AT1G15580.1 in group II, Ath|AT1G80390.1 in group III, Osa|LOCOs09g35870.1 in group IV, Pab|MA478811g0010 in group V, Pab|MA10430040g0010 in group VI, Osa|LOCOs05g09480.1 in group VII, and Atr|AmTrv6.0c12.3350.1 in group VIII. Notably, all five motifs of the Aux/IAA protein were preserved in group I, and the most severe loss of motif was observed in group VI. Interestingly, most species in group VI were lower plants, bryophytes, or ferns, which indicated that the function of Aux/IAA may not have been fully optimized in the early evolutionary stage. Moreover, most of the Aux/IAA proteins in group VI contained motif 1, which may play important roles in the early function of this gene family in plants.

We further explored the evolutionary trajectory of conserved motifs in ARF and Aux/IAA family proteins in detail (Fig. S1). Conserved motifs of ARF family proteins were identified in all 18 representative species, but there was a partial loss of these motifs (Fig. S1a). Among the charophyte species, all 10 motifs in the ARF proteins of *C. atmophyticus*, *C. braunii*, and *M. endlicherianum* were fully retained, while some ARF proteins of *S. muscicola* lacked motifs 3 and 5. Among the moss species, 10 motifs of ARF proteins were fully conserved in *F. antipyretica*, *Entodon seductrix*, *A. agrestis*, and *Anthoceros punctatus*, while some proteins of *F. antipyretica* were missing motifs 1 and 2, and some proteins of *P. patens* were missing motifs 2, 3, 5, and 8. The ARF proteins of *M. polymorpha* had the most motifs missing, with only two motifs (motifs 1 and 5) fully conserved.

The five conserved motifs of the Aux/IAA family proteins had complete deletions in some representative species proteins (Fig. S1b). Motifs 3 and 5 had the same distribution pattern, both of which were completely absent in the charophyte species *C. braunii* and *P. margaritaceum*. Motif 4 was completely lost in the anthocerotophyte *A. agrestis* and fully retained in the charophyte species *C. braunii* and the bryophyte species *M. polymorpha* and *E. seductrix*. Motifs 1 and 2 were relatively conserved compared to motifs 3 − 5.

These results suggest that ARF and Aux/IAA proteins underwent sequence divergence during evolution, but the ARF family proteins were more conserved than the Aux/IAA proteins.

### Identification of duplication types and statistical analysis

**Fig. 4:**
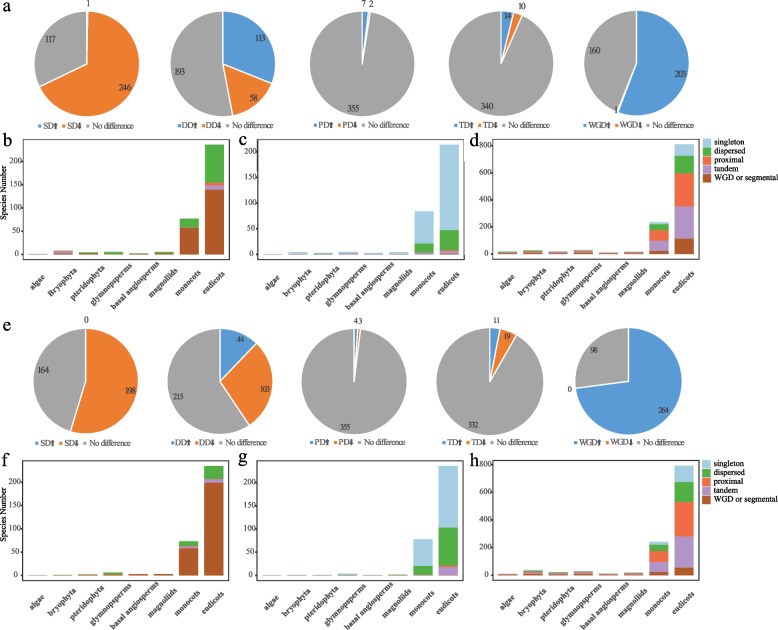
Duplication type and significance analysis for 396 species. **a** The proportion of each significantly enriched or significantly reduced duplicate type of ARF family gene among the total duplicate type. **b** The number of duplicate types in which ARF family genes were significantly enriched in each taxon. **c** The number of duplicate types in which ARF family genes were significantly reduced in each taxon. **d** The number of duplicate species did not change significantly for each taxon of ARF family genes. **e** The proportion of each significantly enriched or significantly reduced duplicate type of Aux/IAA family gene in the total duplicate type. **f** The number of duplicate species in which Aux/IAA family genes were significantly enriched in each taxon. **g** The number of duplicate species in which Aux/IAA family genes were significantly reduced in each taxon. **h** The number of duplicate species did not change significantly for each taxon of the Aux/IAA family genes

To understand the duplication history of ARF and Aux/IAA family genes in plants, we identified five duplicate types in the ARF and Aux/IAA families, namely, singleton, dispersed, proximal, tandem, and whole genome duplication (WGD) or segmental (Fig. 4, Tables S3, S4, S5, S6, S7).

For the singleton gene type (Fig. 4a, e; Table S3), compared to the number of singleton genes at the genomic level, only one species was significantly enriched for singleton genes among the ARF family genes, and none of the species was significantly enriched for singleton genes among the Aux/IAA family genes. A total of 246 and 198 ARF and Aux/IAA family genes, respectively, had significantly lower numbers of singleton genes.

For the dispersed duplication type (Fig. 4a, e; Table S4), compared to the number of dispersed duplications at the genomic level, 113 and 44 species were significantly enriched in dispersed duplications in the ARF and Aux/IAA family genes, respectively. A total of 58 and 103 species had significantly lower numbers of dispersed duplications in the ARF and Aux/IAA family genes, respectively.

For the proximal duplication type (Fig. 4a, e; Table S5), compared to the number of proximal duplications at the genomic level, seven and four species were significantly enriched for proximal duplications in the ARF and Aux/IAA family genes, respectively. A total of two and three species had significantly lower numbers of proximal duplications in the ARF and Aux/IAA family genes, respectively.

For the tandem duplication type (Fig. 4a, e; Table S6), compared to the number of tandem duplications at the genomic level, 14 and 11 species were significantly enriched for tandem duplications in the ARF and Aux/IAA family genes, respectively. A total of 10 and 19 species had significantly lower numbers of tandem duplications in the ARF and Aux/IAA family genes, respectively.

For the WGD type (Fig. 4a, e; Table S7), 203 and 264 species, respectively, were significantly enriched in WGDs of ARF and Aux/IAA family genes compared to the number of WGDs at the genomic level. Only one species had a significantly lower number of WGDs of ARF family genes, while none had a significantly lower number of WGDs of Aux/IAA family genes.

### Comparative analysis of the duplication types in different plant taxa

We further explored the distribution of duplicate types in terms of each plant taxon (Fig. 4b-d, f–h). In algae, a total of one species had significantly enriched singleton genes among the ARF family genes compared to the number of singleton genes at the genomic level (Fig. 4b). However, no species had significantly enriched Aux/IAA family genes among the duplicate genes (Fig. 4f). No species had significantly reduced ARF or Aux/IAA family genes among the duplicated genes compared to the five duplicated genes at the genomic level (Fig. 4c, g).

In bryophytes, compared to the number of dispersed duplications at the genomic level, one species was significantly enriched for dispersed duplications in the ARF and Aux/IAA family genes (Fig. 4b, f). One species was also significantly enriched for proximal duplications in ARF family genes (Fig. 4b). The ARF family genes of four species were significantly enriched for tandem duplications (Fig. 4b). WGD of ARF family genes was significantly enriched in two species (Fig. 4b). Among the four bryophyte species, there were significantly fewer singleton genes among the ARF and Aux/IAA family genes than among the singleton genes at the genome-wide level (Fig. 4c, g).

In ferns, compared to the number of dispersed duplications at the genomic level, two and one species were significantly enriched in dispersed duplications of the ARF and Aux/IAA family genes, respectively (Fig. 4b, f). WGDs of ARF and Aux/IAA family genes were significantly enriched in two and one species, respectively (Fig. 4b, f). Two and one species had significantly lower numbers of singleton genes among the ARF and Aux/IAA family genes, respectively (Fig. 4c, g). One species had a significantly lower number of dispersed duplications of ARF family genes (Fig. 4c).

Among the gymnosperms, compared to the number of dispersed duplications at the genomic level, five and four species were significantly enriched for dispersed duplications in the ARF and Aux/IAA family genes, respectively, (Fig. 4b, f). One species was significantly enriched for proximal duplications and WGDs in the Aux/IAA family genes (Fig. 4f). One species had a significantly lower number of dispersed duplications of the Aux/IAA family genes (Fig. 4g). There were significantly fewer tandem duplications of ARF family genes in these two species than at the genomic level (Fig. 4c).

In basal angiosperms, compared to the number of dispersed duplications at the genomic level, one species was significantly enriched in dispersed duplications of ARF family genes (Fig. 4b). One species was significantly enriched in proximal duplications of the Aux/IAA family genes (Fig. 4f). One and two species were significantly enriched in WGDs of ARF and Aux/IAA family genes, respectively (Fig. 4b, f). Among the ARF and Aux/IAA family genes, three and one species had significantly fewer singleton genes than singleton genes at the genomic level, respectively, (Fig. 4c, g).

Among the magnoliids, a total of three species were significantly enriched in dispersed duplications of ARF family genes compared to the number of dispersed duplications at the genomic level (Fig. 4b). Two and three species were significantly enriched in WGDs of ARF and Aux/IAA family genes, respectively (Fig. 4b, f). Among the ARF and Aux/IAA family genes, four and one species, respectively, had significantly lower numbers of singleton genes (Fig. 4c, g). One species had a significantly lower number of dispersed duplications in the Aux/IAA family genes than at the genomic level (Fig. 4g).

Among the monocots, compared to the number of dispersed duplications at the genomic level, 19 and 10 species were significantly enriched in dispersed duplications of the ARF and Aux/IAA family genes, respectively (Fig. 4b, f). One species was significantly enriched for proximal duplications in the Aux/IAA family of genes (Fig. 4f). One and four species were significantly enriched for tandem duplications in ARF and Aux/IAA family genes, respectively (Fig. 4b, f). A total of 57 and 58 species were significantly enriched in WGDs of ARF and Aux/IAA family genes, respectively (Fig. 4b, f). A total of 63 and 58 ARF and Aux/IAA family genes had significantly lower numbers of singleton genes, respectively (Fig. 4c, g). A total of 18 and 19 species had significantly fewer dispersed duplications of the ARF and Aux/IAA family genes, respectively (Fig. 4c, g). Compared to the number of tandem duplications at the genomic level, three and one species had significantly lower numbers of tandem duplications in the ARF and Aux/IAA family genes, respectively (Fig. 4c, g).

Among the dicots, compared to the number of dispersed duplications at the genomic level 82 and 28 species were significantly enriched in dispersed duplications of the ARF and Aux/IAA family genes, respectively (Fig. 4b, f). Among the ARF and Aux/IAA family genes, six and one species, respectively, were significantly enriched in proximal duplications (Fig. 4b, f). Among the ARF and Aux/IAA family genes, nine and seven species, respectively, were significantly enriched in tandem duplications (Fig. 4b, f). A total of 139 and 199 species were significantly enriched in WGDs (Fig. 4b, f). A total of 167 and 133 species had significantly lower numbers of singleton genes among the ARF and Aux/IAA family genes, respectively (Fig. 4c, g). A total of 39 and 82 species had significantly fewer dispersed duplications of the ARF and Aux/IAA family genes, respectively (Fig. 4c, g). Among the ARF and Aux/IAA family genes, two and three species had significantly lower numbers of proximal genes, respectively (Fig. 4c, g). Five and 18 species had significantly lower numbers of tandem duplications of ARF and Aux/IAA family genes, respectively (Fig. 4c, g). One species had significantly fewer WGDs of ARF family genes than WGDs at the genomic level (Fig. 4c, g).

We found that in the algal plants, the ARF family genes were significantly enriched in the singleton gene type (*P* < 0.05), and the Aux/IAA family genes were not significantly enriched in any of the duplication types (Fig. 4b, f, Table S3, S4, S5, S6, S7). In bryophytes, ARF family genes were significantly enriched in four types (dispersed duplication, proximal duplication, tandem duplication, and WGD), while Aux/IAA family genes were significantly enriched only in the dispersed duplication type (Fig. 4b, f, Table S3, S4, S5, S6, S7). In ferns, ARF and Aux/IAA family genes were significantly enriched in dispersed duplication and WGD (Fig. 4b, f, Table S3, S4, S5, S6, S7). In gymnosperms, ARF family genes were significantly enriched in the dispersed duplication type, and Aux/IAA family genes were significantly enriched in three types (dispersed duplication, proximal duplication, and WGD) (Fig. 4b, f, Table S3, S4, S5, S6, S7).

In basal angiosperms, ARF family genes were significantly enriched in dispersed duplication and WGD, and Aux/IAA family genes were significantly enriched in proximal duplication and WGD (Fig. 4b, f, Table S3, S4, S5, S6, S7). In magnoliids, ARF family genes were significantly enriched in dispersed duplication and WGD, and Aux/IAA family genes were significantly enriched only in the WGD type (Fig. 4b, f, Table S3, S4, S5, S6, S7). In monocots, ARF and Aux/IAA family genes were significantly enriched in three types (dispersed duplication, tandem duplication, and WGD) (Fig. 4b, f, Table S3, S4, S5, S6, S7). Aux/IAA family genes were also enriched in proximal duplication (Fig. 4b, f, Table S3, S4, S5, S6, S7). In dicots, ARF and Aux/IAA family genes were significantly enriched in four types (dispersed duplication, proximal duplication, tandem duplication, and WGD) (Fig. 4b, f, Table S3, S4, S5, S6, S7).

Overall, the mechanisms of expansion of ARF and Aux/IAA family genes differed between lower and higher plants in the species examined here. Among the ARF family genes, bryophytes, ferns, and gymnosperms expanded mainly through dispersed duplication and WGD, while basal angiosperms, magnoliids, monocots, and dicots expanded mainly through WGD. Among the Aux/IAA family genes, bryophytes, ferns, and gymnosperms expanded mainly through dispersed duplication, while basal angiosperms, magnoliids, monocots, and dicots expanded mainly through proximal duplication and WGD.

### Gene expression analysis under different conditions

**Fig. 5:**
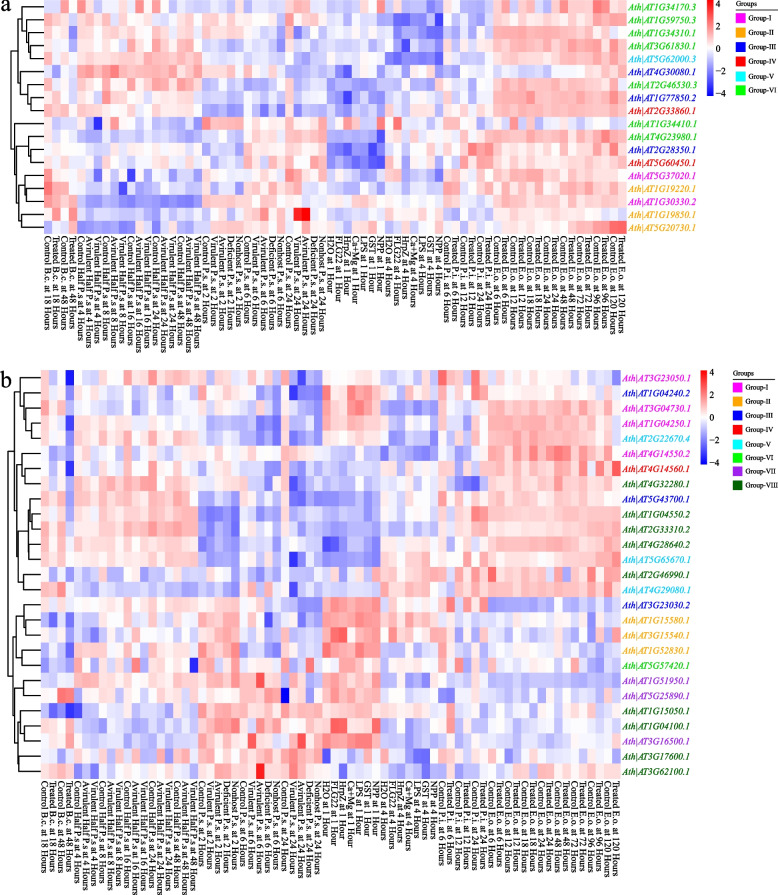
The absolute expression values of ARF and Aux/IAA family genes under various biotic stresses in *A. thaliana*. **a** The expression levels of ARF family genes under various biotic stresses. **b** The expression levels of Aux/IAA family genes under various biotic stresses. The expression data of ARF and Aux/IAA family genes were obtained from the *Arabidopsis* eFP Browser

To explore the potential functions of the ARF and Aux/IAA gene family, we analyzed the expression of the ARF and Aux/IAA gene families in the model plant *A. thaliana* using several expression datasets, including data from different hormone treatments (Fig. S3a, Fig. S5a), different stages of development and tissues (Fig. S3b, Fig. S5b), and a variety of biotic and abiotic stresses (Fig. 5, Fig. S2, S4).

With respect to the expression of the ARF gene family in *A. thaliana*, we found that only two genes, *Ath|AT1G34310.1* located in group VI and *Ath|AT5G62000.3* located in group V, were extremely highly expressed in the majority of the samples. These findings implied that these genes may play central roles in *A. thaliana* growth and development and stress resistance. With respect to the expression of the Aux/IAA gene family in *A. thaliana*, we also found several highly expressed core genes, for example, *Ath|AT3G04730.1* and *Ath|AT3G23050.1* of group I and *Ath|AT2G22670.4* and *Ath|AT5G65670.1* of group V. Unlike for the ARF gene family, the number of highly expressed genes varied depending on the circumstances for the Aux/IAA gene family. For example, *Ath|AT2G33310.2* of group VI was also extremely highly expressed under abiotic stress conditions (Fig. S4).

The differences in the expression of homologous genes were also explored (Fig. S3c, Fig. S5c). Among the ARF gene family, *Cbr|GBG73949* had the highest expression in antheridia and the lowest expression in whole plants (Fig. S3c). The homologous gene *Ath|AT4G30080.1* was highly expressed in tissues such as seeds, stems, and leaves; stamens at the flowering stage; and siliques. For Aux/IAA, *Cbr|GBG75184* had the highest expression in the zygote and lowest expression in the whole plant (Fig. S5c). The homologous gene *Ath|AT4G29080.1* was highly expressed in important whole-plant tissues, such as roots, flowers, leaves, hypocotyls, stems, and seeds. *Cbr|GBG85126* had the highest expression in the zygote and the lowest expression in the archegonia. The homologous gene *Ath|AT5G57420.1* was expressed at low levels in all tissues of *A. thaliana*.

The expression profiles of the Aux/IAA gene family and the ARF gene family were more variable, mainly because the highly expressed genes differed in the different samples. This implied that some genes may have specific functions and play a greater role only under specific conditions. In particular, the number of highly expressed genes increased, which was attributed to differences in the phylogenetic branches under various stresses. This may indicate that the Aux/IAA gene family may require additional members to respond synergistically in the face of various stresses.

### Upstream and downstream gene retrieval and interaction network construction

We used the iGRN database to obtain the upstream and downstream genes of the *A. thaliana* ARF and Aux/IAA gene families (Table S8, S9). Then, regulatory networks were constructed to reveal the regulatory relationships of the genes.

A total of 667 upstream regulatory genes and 2,514 downstream regulatory genes were detected in clade A of the ARF gene family (Fig. S6a, b). Among the upstream regulatory genes, *AT5G37020* in group I had the most upstream regulatory genes (259), while *AT5G20730* in group II had the fewest (76). Among the downstream regulated genes, *AT1G19850* in group II had the most downstream regulated genes (726), while *AT1G30330* in group I had the fewest (308).

A total of 134 upstream genes and 1,837 downstream genes were detected in clade B of the ARF gene family (Fig. S7a, b). Among the upstream genes, *AT1G77850* and *AT4G30080* had the lowest number of upstream genes (40), while *AT2G28350* had the highest number of upstream genes (54). Among the downstream genes, *AT2G28350* had the lowest number of downstream genes (215), while *AT4G30080* had the highest number of downstream genes (1,361). There were significantly more downstream genes than upstream genes in clade B.

**Fig. 6:**
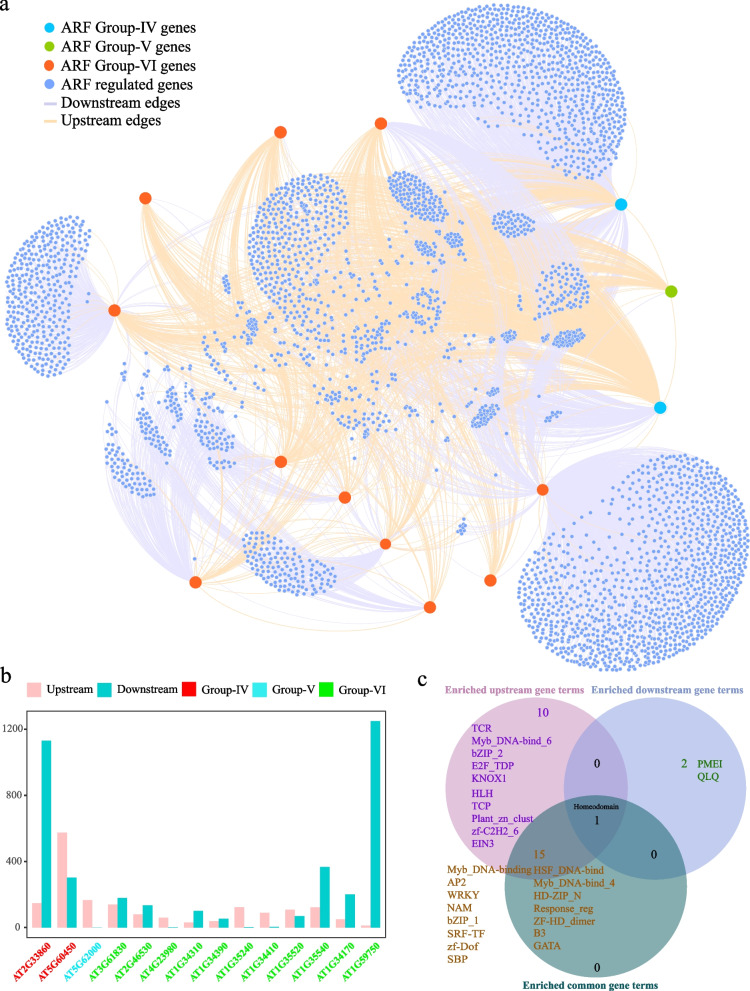
Interaction network of clade C genes of the ARF gene family and their upstream and downstream genes in *A. thaliana*. **a** Construction of the network of clade C genes of the ARF gene family using Gephi software. **b** The number of upstream and downstream genes for each clade C gene of the ARF gene family in the network. **c** The specific and shared terms among the upstream, downstream, and common gene-enriched terms

A total of 1,737 upstream genes and 3,789 downstream genes were detected in clade C of the ARF gene family (Fig. 6a, b). Among the upstream genes, *AT5G60450* in group IV had the most upstream genes (574), while *AT1G59750* in group VI had the least upstream genes (12). Among the downstream genes, *AT1G59750* in group VI had the most downstream genes (1249), while *AT5G62000* in group V had no downstream genes.

**Fig. 7:**
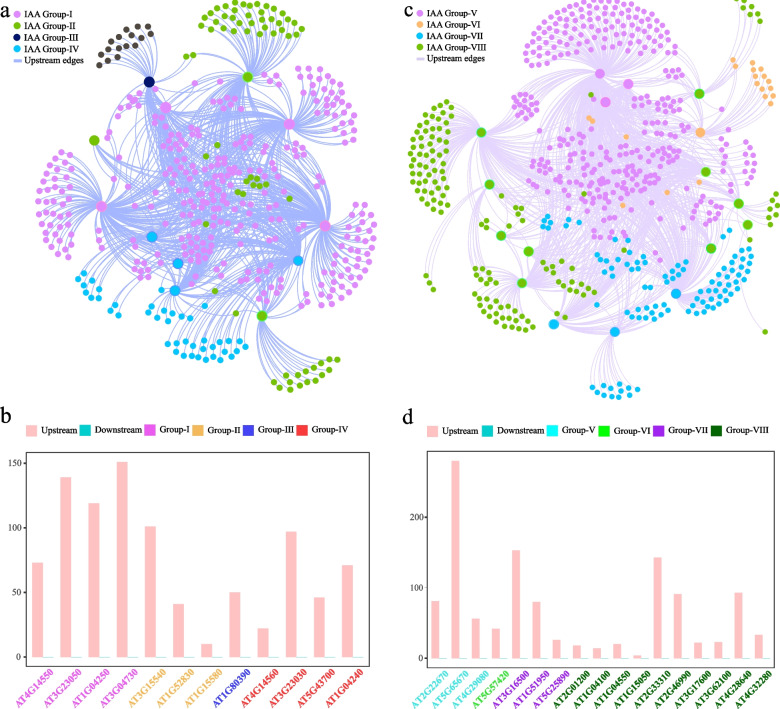
Interaction network of the clade A and B genes of the Aux/IAA gene family and their upstream and downstream genes in *A. thaliana*. **a** Construction of the network among clade A genes of the Aux/IAA gene family using Gephi software. **b** The number of upstream and downstream genes for each clade A gene of the Aux/IAA gene family in the network. **c** Construction of the network among clade B genes of the Aux/IAA gene family using Gephi software. **d** The number of upstream and downstream genes for each clade B gene of the Aux/IAA gene family in the network

A total of 920 upstream genes were detected in clade A of the Aux/IAA gene family, while no downstream genes were found (Fig. 7a, b). Among the upstream genes, *AT3G04730* had the most upstream genes in group I (151), while *AT1G15580* had the fewest upstream genes in group II (10). A total of 1,179 upstream genes were detected in clade B of the Aux/IAA family, while no downstream genes were detected (Fig. 7c, d). Among the upstream genes, *AT5G65670* in group V had the most upstream genes (280), while *AT1G15050* in group VIII had the fewest upstream genes (4).

### Functional annotation and enrichment analysis of upstream and downstream genes

To better understand the functions of the upstream and downstream genes of the *A. thaliana* ARF and Aux/IAA gene families in the networks, a functional enrichment analysis was performed on the upstream and downstream genes involved in each regulatory network (Fig. 6c, Fig. S8a, b). Among the ARF gene family, the upstream genes of clade A were significantly enriched in families such as helix-loop-helix (HLH), teosinte branched1/cincinnata/proliferating cell factor (TCP), and multifunctional mosaic region; downstream genes were significantly enriched in the leucine rich repeat N-terminal_2 family; and upstream and downstream common genes were significantly enriched in families such as QLQ (Fig. S8a, Table S10). The upstream genes of clade B were significantly enriched in families such as WRKY and no apical meristem (NAM), downstream genes were not significantly enriched in any families, and upstream and downstream common genes were significantly enriched in families such as K-boxes (Fig. S8b, Table S11). Clade C upstream genes were significantly enriched in families such as HLH and TCP, and downstream genes were significantly enriched in families such as pectin methylesterase inhibitor and QLQ (Fig. 6c, Table S12).

The common genes upstream and downstream of the ARF family genes in clade A, clade B, and clade C were enriched in the APETALA2 (AP2) and serum response factor-transcription factor (SRF-TF) families, suggesting that they played key roles in the ARF family gene regulatory network.

The enrichment analysis of the upstream genes of the Aux/IAA gene family showed that clade A upstream genes were significantly enriched in the AP2, WRKY, NAM, TCP, SRF-TF, and HLH families (Table S13). Similarly, the clade B upstream genes were enriched in the AP2, WRKY, NAM, TCP, SRF-TF, HLH, GATA, and squamosa-promoter binding protein families (Table S14). In the Aux/IAA family clade A and clade B were enriched in many of the same families, such as the AP2, WRKY, SRF-TF, and TCP gene families. The AP2 family is mainly associated with plant disease resistance (Zhu et al. 2021), the WRKY family is associated with the immune response (Saha et al. 2024), the SRF-TF family is associated with flower development (Ning et al. 2023), and the TCP family is associated with cell differentiation and growth (Viola and Gonzalez 2023). Therefore, the Aux/IAA gene family may mediate plant growth and various stresses through mutual regulation with these gene families. Moreover, the AP2 and SRF-TF families were also enriched in the ARF family gene regulatory network. Therefore, the ARF and Aux/IAA gene families may play important roles in plants by interacting with these two gene families.

## Discussion

The ARF and Aux/IAA gene families are extremely important transcription factor families in the plant auxin signaling pathway that form a complex auxin signaling regulatory network (Woodward and Bartel 2005; Tan et al. 2007; Zhang et al. 2023). The auxin response is dependent on ARF–Aux/IAA interactions mediated by the C-terminal domain (CTD) (Pei et al. 2021). Previous studies of these two plant-specific transcription factor families have investigated only a single or a few species. There has been no comprehensive identification and analysis of the ARF and Aux/IAA gene families in the plant kingdom. As the cost of sequencing decreases, abundant and high-quality genome sequencing data are being released, which provides extremely convenient conditions for large-scale studies of plant hormone-related genes.

In this study, we identified ARF and Aux/IAA family genes in the high-quality genomes of 406 plants, including glaucophytes, prasinodermophytes, rhodophytes, chlorophytes, charophytes, bryophytes, ferns, gymnosperms, basal angiosperms, magnoliids, monocots, and dicots. We found that the earliest algal species in which ARF and Aux/IAA existed were charophyte plants, which helped to determine the origin of the gene family. For the ARF gene family, our result was consistent with a previous analysis of its origin (Gao et al. 2020). However, our large-scale analysis updated the origin of Aux/IAA because we identified Aux/IAA genes in charophytes. This may be due to a previous lack of genomic data for these algal species. Thus, our study revealed that the origins of the ARF and Aux/IAA gene families can be traced back to the charophytes. In addition, among the 406 plants, the numbers of ARF and Aux/IAA family genes were highest in *Dendrocalamus latiflorus* with 129 and 200 genes, respectively, far exceeding the number of genes in the other species. These findings suggested that these two gene families may play important and special roles in this species.

Genes can originate from multiple duplication mechanisms (Song et al. 2016, 2018; Chen et al. 2022, 2023). To understand the origin of the duplication of ARF and Aux/IAA family genes in plants. We performed the large-scale identification and analysis of the duplication origins of ARF and Aux/IAA family genes. We discovered the evolutionary properties of ARF and Aux/IAA family genes in plants. For example, the expansion mechanisms of the ARF and Aux/IAA gene families were different in lower and higher plants. The expansion of the ARF and Aux/IAA gene families in lower plants occurred mainly by dispersed duplication. In higher plants, ARF is mainly quantitatively copied by WGD, while Aux/IAA is mainly copied by proximal duplication and WGD. WGD occurs frequently in plants, and gene families tend to expand significantly with polyploidy events (Song et al. 2020, 2021a; Shen et al. 2022; Zhang et al. 2022). Dispersed duplication is also the main pathway for gene family expansion (Song et al. 2020; Yu et al. 2022a). To our surprise, the expansion of the Aux/IAA gene family was also caused by proximal duplication in some taller plants. This phenomenon reflected the specific evolution of Aux/IAA family genes.

*Arabidopsis thaliana* is the model plant species and the first plant to have its whole-genome sequenced (de 2000; Ondrej 2001). It has long been used as an ideal species for gene function studies. Therefore, we analyzed the expression patterns of the ARF and Aux/IAA gene families in *A. thaliana* under various conditions to gain insights into the functions of these genes in plants. We also constructed an interaction network between the upstream and downstream genes of these two gene families. A functional enrichment analysis revealed that *A. thaliana* ARF and Aux/IAA family upstream and downstream genes were coenriched in the AP2 and SRF-TF families, providing a blueprint for studying their regulatory pathways.

## Conclusion

This study comprehensively identified and analyzed the ARF and Aux/IAA gene families in plant genomes covering the entire plant taxon. The large-scale identification and analysis of ARF and Aux/IAA gene families can help to comprehensively characterize the evolutionary trajectory, structural functions, expansion mechanisms, expression patterns, and regulatory networks of these two families. We found that the ARF and Aux/IAA gene families originated from charophytes. Dispersed duplication was the most common expansion mode of the ARF and Aux/IAA families in bryophytes, ferns, and gymnosperms, while WGD was the most common expansion mode of ARF and Aux/IAA families in basal angiosperms, magnoliids, monocots, and dicots. This study has contributed to the understanding of the molecular evolution of ARF and Aux/IAA family genes. The identification of these genes provides a theoretical basis for understanding hormone-related molecular mechanisms as well as a rich genetic resource for molecular breeding.

## Materials and methods

### Data retrieval

The dataset was obtained from the public database Published Plant Genomes (https://www.plabipd.de/plant_genomes_pa.ep) and other public databases, including the National Center for Biotechnology Information (NCBI) (https://www.ncbi.nlm.nih.gov), Phytozome (https://phytozome-next.jgi.doe.go), China National GeneBank (CNGB) (https://db.cngb.org/search/), the Vegetable Information Resource (TVIR) (http://tvir.bio2db.com) (Yu et al. 2022b), and the Brassicaceae Genome Resource (TBGR) (http://www.tbgr.org.cn) databases (Liu et al. 2022). The latest genomic data (including gff, cds, and protein sequences) of the species were comprehensively collected and collated. The alternative splice sequences were deleted by TBtools (v1.120) (Chen et al. 2020) and Perl scripts to ensure the non-redundancy of the sequences used.

### Identification of family members

The domains of the protein sequences of 406 species were predicted using pfam_scan.pl. The ARF and Aux/IAA family members were extracted from the family IDs of the pfam database (ARF: PF02362, PF06507, and PF02309; Aux/IAA: PF02309) (Wang et al. 2015). To facilitate data processing and analysis, we added the corresponding species abbreviation prefix to the gene id of each original genome(Feng et al. 2024). Python scripts were used to calculate the number of members of the two gene families.

### Phylogenetic analysis and evolutionary trajectory exploration

The protein sequences of 406 species were aligned using MAFFT (v7.475) (Katoh and Standley 2013). FastTree (v2.1) was then used with default parameters to construct phylogenetic trees (Price et al. 2010). According to the topological structure, the phylogenetic trees were divided into different groups. By observing and analyzing the statistics of the different groups, the evolutionary tracks of the two gene families were drawn.

### Conserved motif identification and distribution

The protein sequences of the ARF and IAA family genes of several representative plants were subjected to a motif analysis via a multiple expectation maximization for motif elicitation (MEME) using the default parameters (Bailey et al. 2009). The motif information of the ARF and IAA family genes on the phylogenetic trees was illustrated by the iTOL program (Letunic and Bork 2021).

### Identification of duplication types and statistical analysis

The duplication type of species was identified using MCScanX software (Wang et al. 2012). First, the protein sequences of these species were aligned using the Diamond program with an e-value of 1 × 10^−5^ (Buchfink et al. 2021). Then, the blocks were detected with MCScanX using the default parameters. Finally, the gene duplication types were identified using the duplicate_gene_classifier program of the MCScanX software. A significance analysis of the duplication types for the ARF and IAA family genes compared with whole-genome genes was also conducted using the chi-square test (*p* value < 0.05) (Yu et al. 2022a).

### Gene expression analysis under different conditions

Expression datasets corresponding to various stresses and developmental stages were collected from the *A. thaliana* eFP browser website (Winter et al. 2007). We collected 154 samples from 18 groups under various abiotic stresses, 70 samples from 27 groups under various biotic stresses, 81 samples from 10 groups under various hormones, and 47 samples from different developmental stages of *A. thaliana*. Then, we explored the expression of the ARF and IAA gene families using these biological datasets. The BioLadder platform (https://www.bioladder.cn/web/#/pro/cloud) was used to construct a heatmap based on the clusters of expression data. We also performed ARF and Aux/IAA family gene expression analyses on four tissues of archegonia, antheridia, zygotes, and whole plants of *C. braunii* and compared them with the homologous genes of *A. thaliana*. All expression values were log2 transformed.

### Construction of an upstream and downstream gene retrieval and interaction network

The upstream and downstream genes of the ARF and IAA gene families in *A. thaliana* were identified using the integrated gene regulatory network (iGRN) database, for which the score was ≥ 0.60 (De Clercq et al. 2021). The upstream genes are those that regulate the expression of the ARF and IAA gene families, while the downstream genes are those regulated by the ARF and IAA gene families. The interaction network between family genes and upstream and downstream genes was constructed using Gephi software (v0.9.2) with the continuous graph layout algorithm Yifan Hu (Bastian et al. 2009).

### Functional annotation and enrichment analysis of upstream and downstream genes

The functional annotation of the identified upstream and downstream genes and all the genes of *A. thaliana* was performed using the Pfam database. An enrichment analysis was then conducted using the SciPy package of Python. The *p* values obtained by a significance analysis were further corrected using the Bonferroni method of the R program. A corrected *p* value (q value) < 0.05 and a fold change > 2 were used to define the significant enrichment terms (Yu et al. 2022a). The Evenn platform was used to construct a Venn diagram, which indicated the specific or shared enriched terms associated with the downstream and upstream genes (Chen et al. 2021).

### Supplementary Information

**Additional file 1:Fig. S1.** The distribution of conversed motif related to ARF and Aux/IAA family genes from representative species. (a) The distribution of conversed motif related to ARF family genes from 18 representative species. (b) The distribution of conversed motif related to Aux/IAA family genes from 16 representative species. The eight major plant lineages were represented with different colors. The solid boxes indicate that the motif was presence of in all ARF and Aux/IAA genes in corresponding plant lineages. The dashed boxes suggested that the motif was completely lost or did not exist. The white star indicated the motif was lost in some genes. **Fig. S2.** The absolute expression values of ARF family genes under various abiotic stresses in *Arabidopsis*. The expression data of ARF family genes obtained from the *Arabidopsis* eFP Browser. **Fig. S3.** Comparative expression patterns of ARF family genes between *Arabidopsis thaliana* and *Chara braunii*. (a) The absolute expression values of ARF family genes under various hormone treatments in *A. thaliana*. (b) The absolute expression values of ARF family genes during various developmental stages in different tissues. (c) The expression values of ARF family genes in four tissues (whole plant, archegonia, antheridia, and zygote) of *C. braunii*. The bluer the color is, the lower the expression, and the redder the color is, the higher the expression, and all the expression values are converted by log2. The lines represent homologous relationships. **Fig. S4.** The absolute expression values of Aux/IAA family genes under various abiotic stresses in *Arabidopsis*. The expression data of ARF family genes obtained from the *Arabidopsis* eFP Browser. **Fig. S5.** Comparative expression patterns of Aux/IAA family genes between *Arabidopsis thaliana* and *Chara braunii*. (a) The absolute expression values of Aux/IAA family genes under various hormone treatments in *A. thaliana*. (b) The absolute expression values of Aux/IAA family genes during various developmental stages in different tissues. (c) The expression values of Aux/IAA family genes in four tissues (whole plant, archegonia, antheridia, and zygote) of *C. braunii*. The bluer the color is, the lower the expression, and the redder the color is, the higher the expression, and all the expression values are converted by log2. The lines represent homologous relationships. **Fig. S6.** The interaction network among CladeA genes of ARF family, and their upstream and downstream-regulated genes in *Arabidopsis*. (a) The construction of the network among CladeA genes of ARF family using the Gephi software. (b) The number of upstream and downstream genes for each CladeA gene of ARF family in the network. **Fig. S7.** The interaction network among CladeB genes of ARF family, and their upstream and downstream-regulated genes in *Arabidopsis*. (a) The construction of the network among CladeB genes of ARF family using the Gephi software. (b) The number of upstream and downstream genes for each CladeB gene of ARF family in the network. **Fig. S8.** The specific and shared terms among upstream, downstream, and common gene enriched terms among CladeA and CladeB genes of ARF family. (a) The specific and shared terms among upstream, downstream, and common gene enriched terms among CladeA genes of ARF family. (b) The specific and shared terms among upstream, downstream, and common gene enriched terms among CladeB genes of ARF family.

**Additional file 2:Table S1.** The classification and genomic data source of 406 species. **Table S2.** The number of 406 species in different taxa used in this study. **Table S3.** The statistics and significance analysis of the number of ARF and Aux/IAA singleton duplication genes in 396 species with *P*-value<0.05. **Table S4.** The statistics and significance analysis of the number of ARF and Aux/IAA dispersed duplication genes in 396 species with *P*-value<0.05. **Table S5.** The statistics and significance analysis of the number of ARF and Aux/IAA proximal duplication genes in 396 species with *P*-value<0.05. **Table S6.** The statistics and significance analysis of the number of ARF and Aux/IAA tandem duplication genes in 396 species with *P*-value<0.05. **Table S7.** The statistics and significance analysis of the number of ARF and Aux/IAA WGD/segmental duplication genes in 396 species with *P*-value<0.05. **Table S8.** The number of up-and downstream genes related to ARF in the network of *A. thaliana*. **Table S9.** The number of up-and downstream genes related to Aux/IAA in the network of *A. thaliana*. **Table S10.** The functional enrichment analysis of target genes related to CladA of ARF family in the *Arabidopsis*. The enriched terms based on Pfam annotations with *q*-value < 0.05. **Table S11.** The functional enrichment analysis of target genes related to CladB of ARF family in the *Arabidopsis*. The enriched terms based on Pfam annotations with *q*-value < 0.05. **Table S12.** The functional enrichment analysis of target genes related to CladC of ARF family in the *Arabidopsis*. The enriched terms based on Pfam annotations with *q*-value < 0.05. **Table S13.** The functional enrichment analysis of target genes related to CladA of Aux/IAA family in the *Arabidopsis*. The enriched terms based on Pfam annotations with *q*-value < 0.05. **Table S14.** The functional enrichment analysis of target genes related to CladB of Aux/IAA family in the *Arabidopsis*. The enriched terms based on Pfam annotations with *q*-value < 0.05.

## Data Availability

All relevant data that support the findings of this study are available from the corresponding author upon request.

## Abbreviations

AFB: ABA-responsive fba domain-containing protein
AP2: APETALA2
ARF: Auxin response factor
Aux/IAA: Auxin/indole-3-acetic acid
AuxREs: Auxin response elements
HLH: Helix-loop-helix
LRRNT_2: Leucine rich repeat N-terminal_2
MFMR: Multifunctional mosaic region
NAM: No apical meristem
PMEI: Pectin methylesterase inhibitor
SBP: Squamosa-promoter binding protein
SRF-TF: Serum response factor-transcription factor
TCP: Teosinte branched1/cincinnata/proliferating cell factor
TPL: TOPLESS
WGD: Whole genome duplication
