# Supplementary material for: Large-scale analysis of the ARF and Aux/IAA gene families in 406 horticultural and other plants
Source: Mol Hortic. 2024 Apr 9;4:13. doi: 10.1186/s43897-024-00090-7 (PMC11003162; doi:10.1186/s43897-024-00090-7)
Supplement: Supplementary file 1 — Additional file 1:Fig. S1. The distribution of conversed motif related to ARF and Aux/IAA family genes from representative species. (a) The distribution of conversed motif related to ARF family genes from 18 representative species. (b) The distribution of conversed motif related to Aux/IAA family genes from 16 representative species. The eight major plant lineages were represented with different colors. The solid boxes indicate that the motif was presence of in all ARF and Aux/IAA genes in corresponding plant lineages. The dashed boxes suggested that the motif was completely lost or did not exist. The white star indicated the motif was lost in some genes. Fig. S2. The absolute expression values of ARF family genes under various abiotic stresses in Arabidopsis. The expression data of ARF family genes obtained from the Arabidopsis eFP Browser. Fig. S3. Comparative expression patterns of ARF family genes between Arabidopsis thaliana and Chara braunii. (a) The absolute expression values of ARF family genes under various hormone treatments in A. thaliana. (b) The absolute expression values of ARF family genes during various developmental stages in different tissues. (c) The expression values of ARF family genes in four tissues (whole plant, archegonia, antheridia, and zygote) of C. braunii. The bluer the color is, the lower the expression, and the redder the color is, the higher the expression, and all the expression values are converted by log2. The lines represent homologous relationships. Fig. S4. The absolute expression values of Aux/IAA family genes under various abiotic stresses in Arabidopsis. The expression data of ARF family genes obtained from the Arabidopsis eFP Browser. Fig. S5. Comparative expression patterns of Aux/IAA family genes between Arabidopsis thaliana and Chara braunii. (a) The absolute expression values of Aux/IAA family genes under various hormone treatments in A. thaliana. (b) The absolute expression values of Aux/IAA family genes during various [file 43897_2024_90_MOESM1_ESM.pdf]

Supplementary Figures 1-8

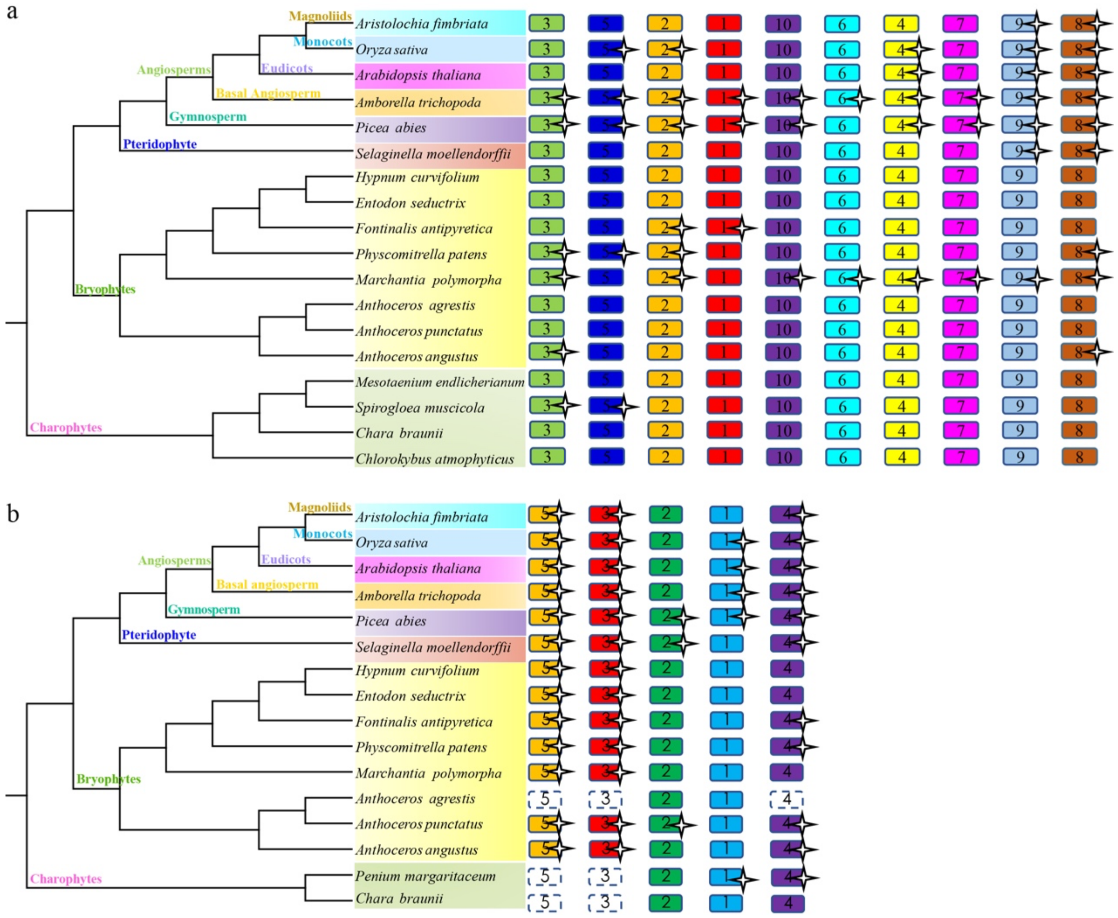

Fig. S1. The distribution of conversed motif related to ARF and Aux/IAA family genes from representative species. (a) The distribution of conversed motif related to ARF family genes from 18 representative species. (b) The distribution of conversed motif related to Aux/IAA family genes from 16 representative species. The eight major plant lineages were represented with different colors. The solid boxes indicate that the motif was presence of in all ARF and Aux/IAA genes in corresponding plant lineages. The dashed boxes suggested that the motif was completely lost or did not exist. The white star indicated the motif was lost in some genes.

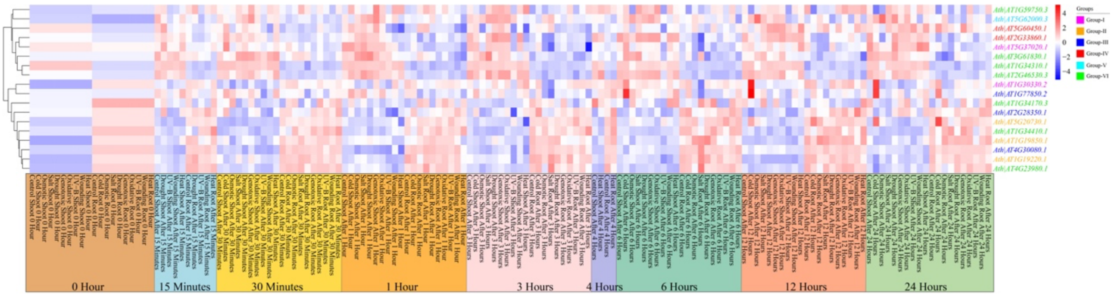

Fig. S2. The absolute expression values of ARF family genes under various abiotic stresses in *Arabidopsis*. The expression data of ARF family genes obtained from the *Arabidopsis* eFP Browser.

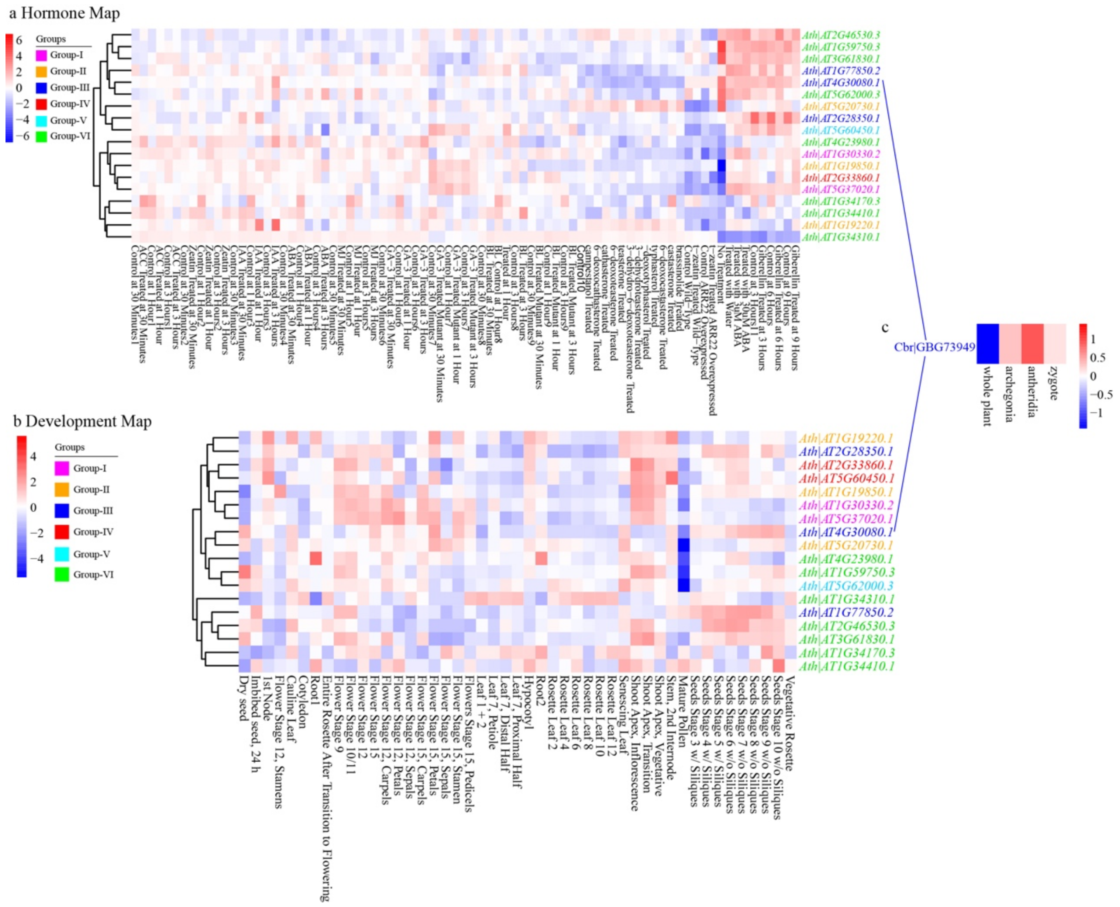

Fig. S3. Comparative expression patterns of ARF family genes between *Arabidopsis thaliana* and *Chara braunii*. (a) The absolute expression values of ARF family genes under various hormone treatments in *A. thaliana*. (b) The absolute expression values of ARF family genes during various developmental stages in different tissues. (c) The expression values of ARF family genes in four tissues (whole plant, archegonia, antheridia, and zygote) of *C. braunii.* The bluer the color is, the lower the expression, and the redder the color is, the higher the expression, and all the expression values are converted by log2. The lines represent homologous relationships.

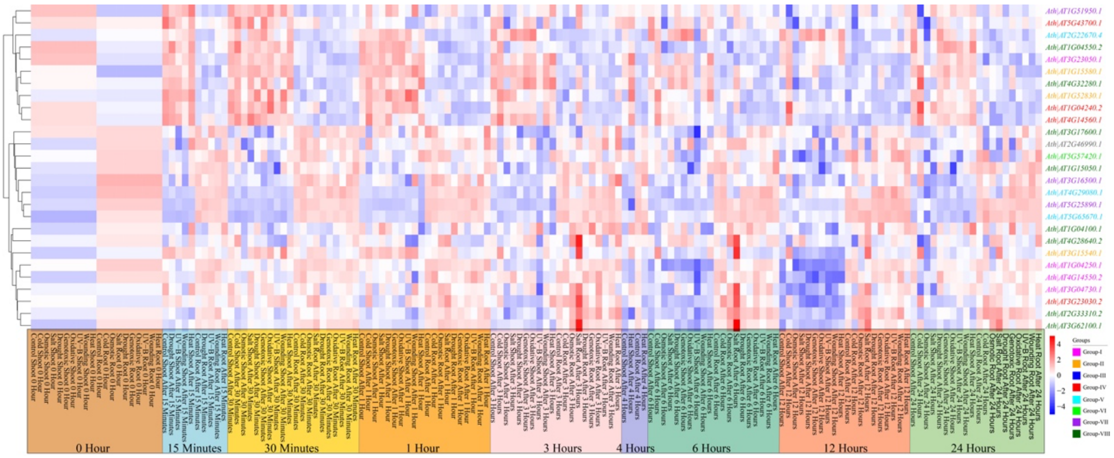

Fig. S4. The absolute expression values of Aux/IAA family genes under various abiotic stresses in *Arabidopsis*. The expression data of ARF family genes obtained from the *Arabidopsis* eFP Browser.

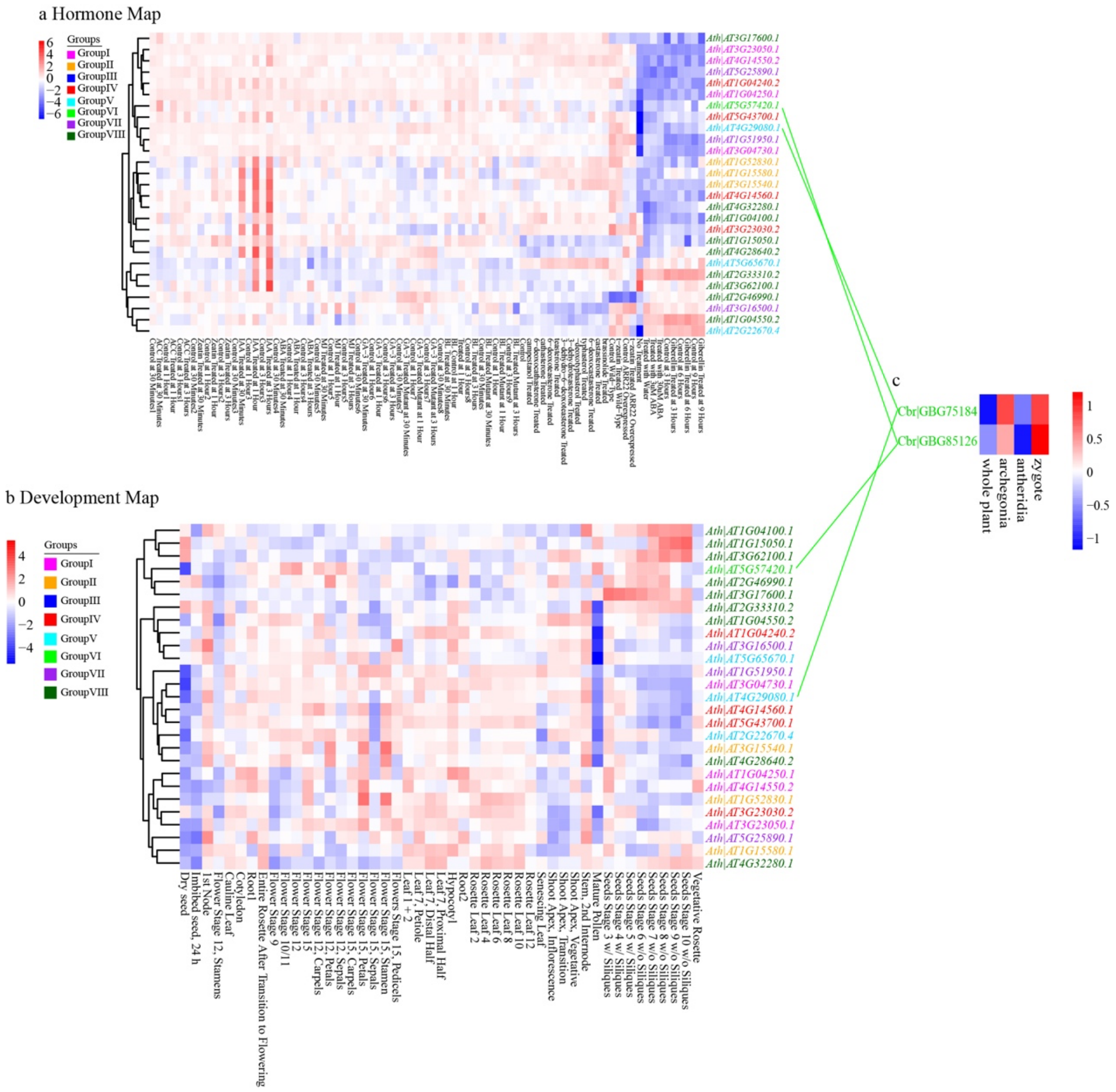

Fig. S5. Comparative expression patterns of Aux/IAA family genes between *Arabidopsis thaliana* and *Chara braunii*. (a) The absolute expression values of Aux/IAA family genes under various hormone treatments in *A. thaliana*. (b) The absolute expression values of Aux/IAA family genes during various developmental stages in different tissues. (c) The expression values of Aux/IAA family genes in four tissues (whole plant, archegonia, antheridia, and zygote) of *C. braunii.* The bluer the color is, the lower the expression, and the redder the color is, the higher the expression, and all the expression values are converted by log2. The lines represent homologous relationships.

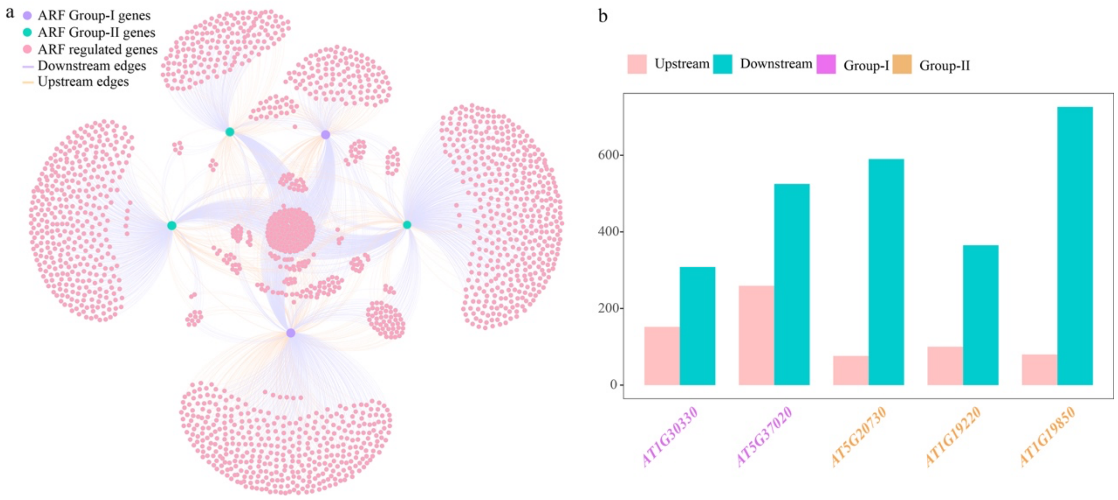

Fig. S6. The interaction network among CladeA genes of ARF family, and their upstream and downstream-regulated genes in *Arabidopsis*. (a) The construction of the network among CladeA genes of ARF family using the Gephi software. (b) The number of upstream and downstream genes for each CladeA gene of ARF family in the network.

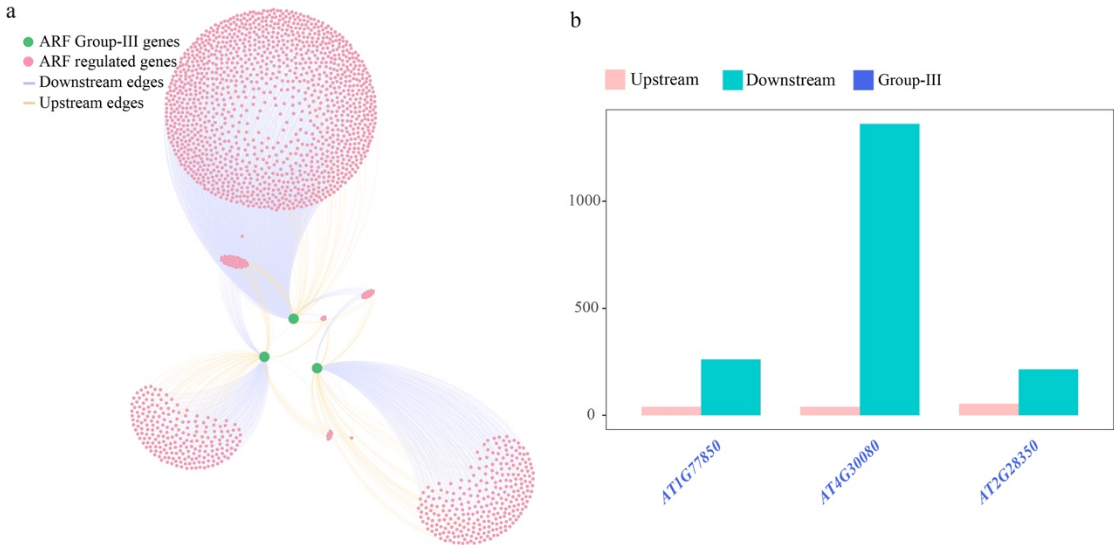

Fig. S7. The interaction network among CladeB genes of ARF family, and their upstream and downstream-regulated genes in *Arabidopsis*. (a) The construction of the network among CladeB genes of ARF family using the Gephi software. (b) The number of upstream and downstream genes for each CladeB gene of ARF family in the network.

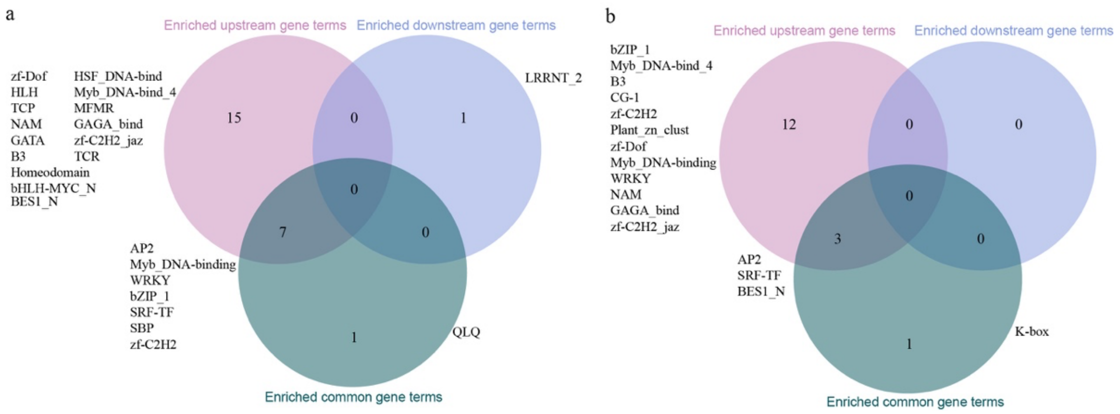

Fig. S8. The specific and shared terms among upstream, downstream, and common gene enriched terms among CladeA and CladeB genes of ARF family. (a) The specific and shared terms among upstream, downstream, and common gene enriched terms among CladeA genes of ARF family. (b) The specific and shared terms among upstream, downstream, and common gene enriched terms among CladeB genes of ARF family.
